# An Exploration of Alkaline Degumming in the Printing and Dyeing Process of Silk Georgette

**DOI:** 10.3390/polym16202926

**Published:** 2024-10-18

**Authors:** Huihui Wu, Jiali Zhou, Panpan Zhu, Jing Li, Yufeng Li

**Affiliations:** 1 Pan Tianshou College of Architecture, Art and Design, Ningbo University, Ningbo 315211, China; wuhuihui@nbu.edu.cn (H.W.); 15857579642@163.com (J.Z.); 2 International Institute of Silk, Zhejiang Sci-Tech University, Hangzhou 310018, China; zhupanpan99322@gmail.com; 3 Fashion Department of Ningbo University-University of Angers Joint Institute, Ningbo University, Ningbo 315211, China; lj7980062@163.com

**Keywords:** alkaline printing, silk georgette, alkaline boiling degumming, alkaline steaming degumming, pattern detail

## Abstract

Alkali printing was one of the traditional techniques employed for printing on silk georgette in ancient China. This study investigates two degumming methods in alkaline printing processes, namely alkaline boiling and alkaline steaming, based on the principles of Tang Dynasty alkaline printing techniques. The effects of slaked lime concentration, steam temperature, and steam duration on the degumming rate of silk georgette are studied. Alkaline boiling is found to be rapid and effective, achieving a degumming rate of 27% at 80 °C in 30 min, whereas alkaline steaming requires a prolonged process with a maximum degumming rate of less than 20% before the water reaches its boiling point. Additionally, the differences in dyeing effects at various degumming rates, and the variations in pattern clarity and detail under alkaline steaming, were compared. Although both degumming methods can achieve the desired amount of degumming rate through process control, alkaline steaming allows for integration with methods like screen printing and rotary printing, offering better control over pattern freedom and detail. The combination of these two processes can further expand the artistic expression and application of traditional alkaline printing techniques in contemporary silk degumming printing.

## 1. Introduction

Alkali printing was one of the traditional methods used for printing on silk georgette in ancient China. The earliest evidence of this technique is found in four Tang Dynasty silk prints excavated from the Astana graves in Xinjiang. It was first introduced and temporarily named the “alkali printing method” in a research report by Wu Min [1]. Alkali printing is a method that employs alkaline substances to dissolve silk sericin and inhibit dye absorption on certain dyes, enabling either decolorization or dye resistance. The process involves preparing a printing paste from strongly alkaline materials such as plant ashes or lime, which is then applied to raw silk fabric [2]. Sericin is water-soluble and easily soluble in hot water, acid, and alkali solutions [3]. This causes the silk in the printed areas to swell and degum, resulting in patterns with a glossy finish similar to that of processed silk. Without dyeing, the raw silk shows patterns in its natural color. Sericin content affects dyeing rate. After dyeing, the variations in dye uptake between raw and processed silk create different shades of color [4]. The printed areas exhibit soft, fluffy fibers with significant gloss, while the raw silk regions remain firm, lightweight, and transparent [5]. This technique allows for the presentation of diverse colors and textures within the same piece of raw silk fabric, offering a rich and unique three-dimensional printing style that aligns with contemporary fashion esthetics and personal creativity. This technique has neither been documented in the literature nor passed down through subsequent generations. It is considered an innovation of the silk-weaving and dyeing artisans of the Tang Dynasty, holding significant traditional craft and cultural value [6].

In “alkali printing”, the partial degumming of raw silk is crucial for revealing patterns, and factors such as the method and degree of degumming significantly influence the pattern effects. Researchers both domestically and internationally have developed numerous methods for removing sericin from silk, including soap boiling, alkali boiling, acid boiling, organic amine treatments, enzyme treatments, and composite treatments [7]. However, most of these methods focus on the overall degumming of the silk or silk textiles, rather than partial degumming and pattern coloration [8]. Alkali boiling degumming printing (hereinafter abbreviated as the “alkaline boiling” method), combined with traditional anti-staining technique such as tie-dyeing and batik, achieves varied printing colors through different levels of degumming. This technique produces patterns that showcase the unique esthetic of traditional dyeing and printing craftsmanship. However, it is also constrained by the style of the technique, resulting in relatively simple and abstract patterns with limited flexibility and freedom [9]. In the field of textile printing, the depth of print color and the clarity of pattern contours are key indicators of the appearance of silk-printed fabrics [10]. To ensure rich color and intricate patterns, it is essential to determine the appropriate level of partial degumming when processing solid color patterns and to minimize the bleeding of pattern contours during degumming and printing [11]. Compared to alkali boiling, alkali steaming degumming printing (hereinafter abbreviated as the “alkaline steaming” method) offers greater control in expressing pattern freedom and detail.

This study utilizes slaked lime as an alkaline degumming agent to compare the effects of two different degumming methods—alkali boiling and alkali steaming—on the degumming rate of silk georgette. The investigation focuses on the impact of factors such as slaked lime concentration, steam temperature, and steam duration on the degumming rate of silk. It also examines the differences in the dyeing effects of silk georgette at various degumming rates, as well as the changes in pattern clarity and detail under alkali steaming. By employing a scientific and quantitative approach, this research aims to establish a comparative framework for alkali printing processes suited to different visual effects. The goal is to explore how variations in degumming methods—specifically, alkali boiling versus alkali steaming—can achieve greater freedom in pattern expression and detail. This will potentially expand the artistic expression and application of traditional Chinese alkali printing techniques in contemporary silk degumming and printing practices.

## 2. Experiment

### 2.1. Materials, Reagents, and Equipment

The fabric used was silk georgette, made from raw mulberry silk treated by low-temperature swelling. The silk had a filament count of 20/22 denier and a mass of 34 g/m^2^, with 100% mulberry silk, and a plain weave, sourced from Zhongzhaoji Jinli Co., Ltd., Huzhou, China. Slaked lime (analytical grade, from National Pharmaceutical Group Chemical Reagents Co., Ltd., Shanghai, China), wheat starch (food grade, from Baoding Brewing Co., Ltd., Shanghai, China), urea (analytical grade, from National Pharmaceutical Group Chemical Reagents Co., Ltd., Shanghai, China), soda ash (food grade, from Yinuo Biotechnology Co., Ltd., Hangzhou, China), distilled water (from Herxin Electromechanical Co., Ltd., Suzhou, China), and pomegranate peel (commercially purchased) were also used. A pH meter (Dongguan Wanchuang Electronics Co., Ltd., Guangdong, China), a high-precision electronic balance (Youkewit Electronic Technology Co., Ltd., Kunshan, China), a steam box (ZQB400-S273, Boss Electric Co., Ltd., Hangzhou, China), a 101-1B constant temperature electric air drying oven (Xingchen Instrument Factory, Yuyao, China), an AS-12/24 room temperature oscillating dyeing sample machine (Jingke Textile Printing and Dyeing Equipment Co., Ltd., Foshan, China), a ZEISS Sigma 300 scanning electron microscope (Berlin, Germany), an XSP-16A optical microscope(Yongxin Optical Co., Ltd., Nanjing, China) and a CS-5960GX spectrophotometer (3nh Technology Co., Ltd., Shenzhen, China) were also used. 

### 2.2. Experimental Methods

#### 2.2.1. Preparation of Alkaline Solution

Select a specified amount of slaked lime and dissolve it in room temperature distilled water. After the liquid becomes clear, decant the upper clear solution. For the preparation of saturated lime water: at room temperature, add more than 0.2 g of slaked lime to 100 mL of distilled water, stir thoroughly to dissolve, and let it stand overnight. Afterward, a lime precipitate will form at the bottom of the solution; decant the clear upper liquid to obtain a saturated lime alkaline solution.

#### 2.2.2. Preparation of Alkaline Paste

Weigh 5% urea and varying amounts of wheat starch and dissolve them in a fixed concentration of lime water. Heat the mixture in a water bath until it nears boiling, then cook for 5 min while stirring continuously. This produces a uniform, granule-free, semi-transparent alkaline printing paste, which is then set aside for use.

#### 2.2.3. Alkali Boiling Degumming Printing (Namely, Alkaline Boiling)

Weigh and record the mass of the silk georgette fabrics (cut to 20 cm × 20 cm) using an electronic balance. Fold each piece of fabric into four layers and place it between two 5 cm × 5 cm high-strength resin boards, securing it with a constant clamping force using a G-clamp. Immerse the clamped fabric in alkali water with different pH values at a bath ratio of 1:80. Perform the degumming process at various temperatures and times, then repeatedly wash with distilled water until the wash solution reaches a neutral pH. Finally, dry the degummed fabric in a 95 °C oven for 2 h, and after equilibrating overnight, weigh and store it for performance testing. This test is repeated three times to determine the degumming rate of the silk georgette.

#### 2.2.4. Alkali Steaming Degumming Printing (Namely, Alkaline Steaming)

Weigh and record the mass of the fabric and fix it to the printing plate. Use a plain mesh screen with a monofilament diameter of 106 μm (150 mesh, 200 mesh) to print large square patterns and “cross” fine line patterns, each 10 cm × 10 cm in size. Place the printed fabric in a high-temperature steam oven and process it at specific temperatures and times. Wash the fabric with warm water (40–55 °C), then rinse repeatedly with cold water until the paste is removed and the water used for cleaning becomes neutral. Finally, dry the fabric, weigh it, and store it after equilibration overnight. The large square pattern test is repeated three times to assess the degumming rate of the silk georgette.

#### 2.2.5. Dyeing

Prepare the dye solution by washing fresh pomegranate peels, drying them in an oven, and then grinding them into a powder using a mill. Create a 6% pomegranate peel dye solution by boiling the powder in water at 100 °C for 30 min, replenishing the distilled water lost to evaporation as needed. Once cooled, filter the dye solution using a sieve, adjust the pH to 9 with soda ash, and set aside. For dyeing, use the pomegranate peel dye solution at a bath ratio of 1:30, dyeing at 60 °C for 60 min [12]. After dyeing, remove the samples, wash them with water, and dry them. This process is conducted using a computer-controlled room-temperature dyeing sample machine.

### 2.3. Testing Methods

#### 2.3.1. Degumming Rate Calculation

Weigh the fabric accurately using an electronic balance and calculate the degumming rate with the following formula:

R (%) = (*m*_1_ − *m*_2_)/*m*_1_ × 100%

where *m*_1_ is the mass of the silk georgette before degumming treatment and *m*_2_ is the mass of the silk georgette after drying post-degumming [13].

#### 2.3.2. SEM Imaging

Use the ZEISS Sigma 300 scanning electron microscope (Berlin, Germany) to observe the surface morphology and structure of the fabric before and after printing paste application.

#### 2.3.3. Apparent Color Depth

Measure the K/S values on the front and back sides of the fabric using the CS-5960GX spectrophotometer. Fold the fabric into four layers, use a D65 light source, and set the viewing angle to 10°. Measure each sample at different locations five times and calculate the average value [14].

#### 2.3.4. Pattern Clarity

Photograph the printed line patterns, with a width of 1000 μm, using an optical microscope. Measure the line widths using Nano Measurer software (Visual Basic 6.0), conducting 30 measurements, and calculate the average line width [15]. Evaluate the pattern clarity by comparing the measured line width to the original line width.

## 3. Results and Discussion

### 3.1. Effect of Slaked Lime Quantity on Degumming Rate of Silk Georgette

**Figure 1:**
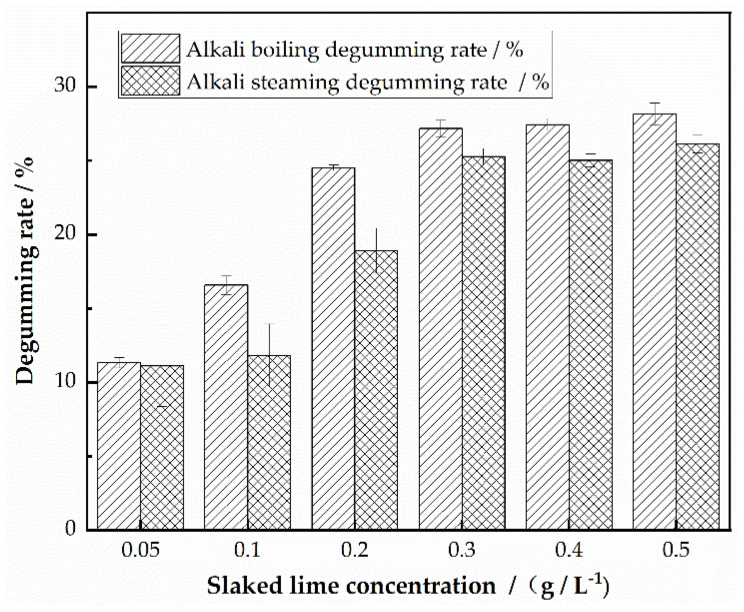
Effect of slaked lime concentration on degumming of silk georgette.

Using the methods described in Section 2.2.3 and Section 2.2.4, the impact of slaked lime quantity on fabric degumming efficiency was studied under fixed conditions: alkali boiling (80 °C, 30 min, bath ratio 1:80) and alkali steaming (100 °C, 120 min). Three samples were tested for each condition, and the average degumming rate and standard deviation were calculated. The results are presented in Table 1, and Figure 1 provides a visual bar chart of the experimental results.

At room temperature (25 °C), with a slaked lime concentration of 0.05%, the solution pH was 11. Increasing the concentration to above 2.0 g/L led to the saturation of the solution, with the pH stabilizing around 12.50. Figure 1 shows that both alkali boiling and alkali steaming result in a continuous increase in degumming rate with the rising pH of the alkaline solution. Additionally, alkali boiling consistently achieves a slightly higher delignification rate compared to alkali steaming. When the slaked lime solution reached saturation, the degumming rates stabilized, with alkali boiling achieving approximately 27% and alkali steaming about 25%. This difference is attributed to the fact that alkali boiling can more effectively surround and remove sericin from the silk surface, while alkali steaming relies on the gradual release of alkali agents from the paste through steam, making the degumming process slower and less efficient [16]. It is evident that the degumming rate of silk georgette is directly related to the amount of slaked lime used. Once saturation is reached, the alkalinity does not cause excessive degumming or damage to the silk fabric. Therefore, subsequent experiments used saturated lime water.

### 3.2. Effect of Temperature on Degumming Rate of Silk Georgette

Temperature plays a crucial role in alkaline degumming, but the sensitivity to temperature varies significantly between alkali boiling and alkali steaming. To analyze this effect, comparative experiments were conducted using both methods. Following the procedures outlined in Section 2.2.3, a 0.3% slaked lime saturated solution was used for alkali boiling at a bath ratio of 1:80 for 30 min to observe the impact of different boiling temperatures on the degumming rate. For alkali steaming, the method in Section 2.2.4 was adopted, involving a 0.3% slaked lime solution with 10% starch being used to apply a paste, and steaming was performed at different temperatures for 120 min to study the effect of steaming temperature on the degumming rate.

**Figure 2:**
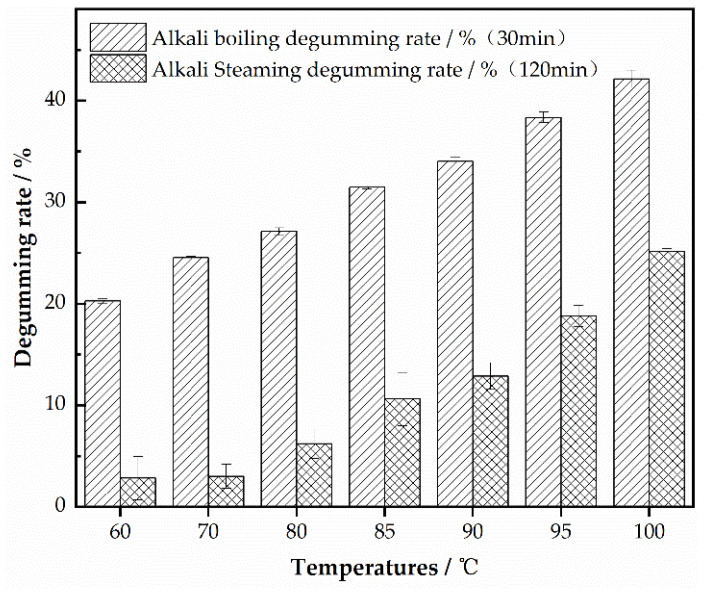
Effect of different temperatures on degumming of silk georgette.

Table 2 illustrates a significant difference in temperature sensitivity between alkali boiling and alkali steaming. At 60 °C, the degumming rate for alkali boiling reached 20%, while for alkali steaming it was less than 3%. This disparity is due to the more effective contact between sericin and the solution in alkali boiling, whereas in alkali steaming, the water does not quite reach its boiling point, resulting in limited steam production [17]. Figure 2 clearly shows that as the temperature increased, the degumming rate for alkali boiling approached 27% at 80 °C, nearing complete degumming. Though further temperature increases began to damage the silk fibers, at 100 °C, the weight loss of the fabric reached 42%, with noticeable damage. In contrast, alkali steaming showed a more gradual response to temperature changes. Even at 100 °C, the degumming rate only reached around 25%, without causing damage to the fabric. It is evident that under the same temperature conditions, alkali boiling at 30 min results in a significantly higher rate of sericin removal compared to alkali steaming at 120 min. During alkali steaming, after the water reaches boiling, steaming for 2 h results in a degumming rate of only around 25%. For subsequent experiments, unless otherwise specified, alkali steaming would be conducted at a steam temperature of 100 °C.

### 3.3. Effect of Time on the Degumming Rate of Silk Georgette

To investigate the effect of time on the degumming rate, the following methods were used: Using the method described in Section 2.2.3, a 0.3% slaked lime saturated solution was prepared, and degumming was conducted at a bath ratio of 1:80 and a fixed temperature of 80 °C. The impact of different boiling times on the degumming rate of silk georgette was observed. For alkali steaming, as outlined in Section 2.2.4, a 0.3% slaked lime saturated solution with 10% starch was used to prepare the alkaline printing paste. A 10 cm × 10 cm square pattern was applied by scraping the paste onto the fabric. The effect of different steaming durations on the degumming rate was assessed at a fixed steaming temperature of 100 °C. Three samples were tested for each condition, and the average degumming rate and standard deviation were calculated. The results are presented in Table 3.

**Figure 3:**
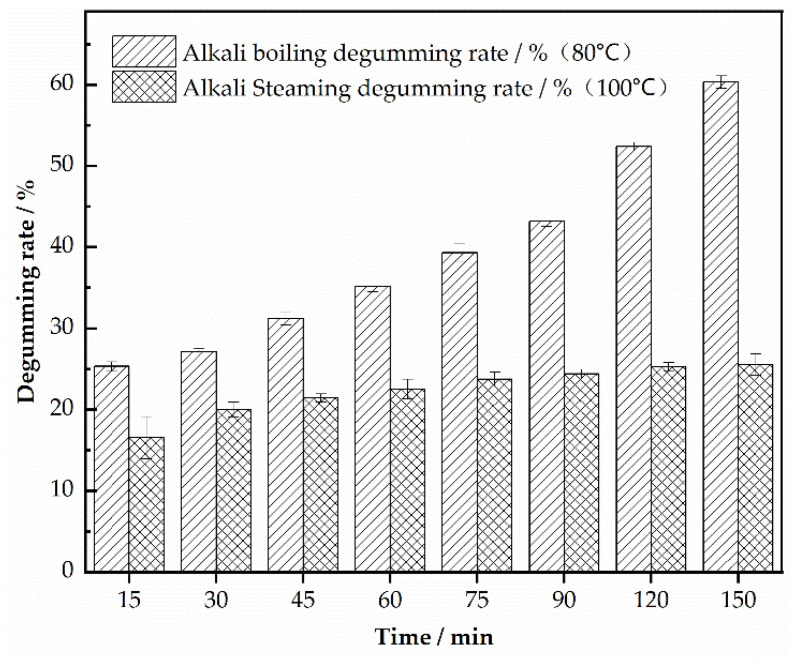
Effect of different steam time on degumming of silk georgette.

Figure 3 demonstrates the effect of different steam times on the degumming of silk georgette. As shown in Figure 3, under saturated conditions of slaked lime solution, the degumming rate of silk georgette increases with time in both alkali boiling and alkali steaming. In alkali boiling, at 15 min, the degumming rate reached 25.34%. With an increase to 30 min, it reached 27.12%, indicating nearly complete degumming. However, further extending the treatment time begins to damage the silk fibers and cause fabric degradation. In contrast, for alkali steaming, the degumming rate shows a gradual increase over time, from 16.54% at 15 min to around 25.54% at 120 min, after which it stabilizes. Additional time beyond this point does not significantly alter the degumming rate.

**Figure 4:**
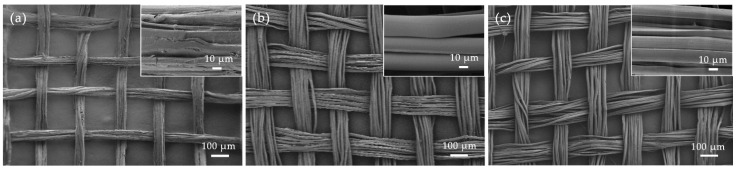
Silk yarn monofilament and its fabric surface morphology under alkali boiling and steaming degumming. (**a**) Not degummed; (**b**) degummed from alkali boiling (80 °C, 30 min); (**c**) degummed from alkali steaming (100 °C, 120 min).

Previous studies [18] indicate that sericin content in silk cocoons is approximately 20–30%, with a loss of 5–7% during the reeling process. Additionally, the low-temperature soaking of silk can lead to further sericin loss, so the actual sericin content in silk georgette is around 25%. At 100 °C, steam with high-energy water molecules and strong permeability breaks protein–protein hydrogen bonds, leading to the gradual hydrolysis of sericin and its separation from the silk fibroin [19]. Furthermore, the high-concentration alkaline printing paste absorbs moisture during steaming, which promotes further hydrolysis of silk proteins. This is corroborated by the SEM images in Figure 4, where nearly complete removal of sericin is observed at a degumming rate of around 25%.

### 3.4. Surface Morphology of Silk Georgette under Different Degumming Treatments

The surface morphology and structure of silk georgette under different degumming conditions were further examined using scanning electron microscopy (SEM). Figure 4a shows the surface of untreated silk georgette. The fibers appear rough, with sericin tightly bonding multiple fibroin strands together [20]. There are noticeable gaps between the warp and weft yarns. Figure 4b displays the surface morphology of silk georgette after degumming with high-temperature alkali boiling (using saturated lime water, in a bath ratio of 1:80, at 80 °C for 30 min). Figure 4c illustrates the surface of silk georgette treated with alkali steaming (using saturated lime water paste, at a steam temperature of 100 °C, for 120 min). In both degumming methods—alkali boiling and alkali steaming—a noticeable separation in the silk fibers is observed. The fiber surfaces are smooth with no remaining sericin, and the fibers exhibit fine longitudinal striations due to the original fiber structure. The fabric structure becomes looser, and the softness is increased [21]. Overall, there are no significant differences in the surface morphology of silk georgette between the alkali boiling and alkali steaming.

### 3.5. Infrared Spectroscopic Analysis of Degummed Fibers

The significant differences in the transmittance and absorption peaks of silk with varying degrees of degumming are primarily attributed to the profound impact of the degumming process on the molecular structure and chemical environment of silk. The Silk-I crystalline structure is a metastable arrangement formed by the stacking of silk fibroin chains in an α-type molecular conformation [22]. Following treatments such as moisture, heat, stress, and polar solvents, Silk-I (α-helix) can transform into Silk-II (β-sheet). As the degree of degumming increases, the water-soluble and unstable Silk-I (α-helix) gradually diminishes or disappears, while the stable and water-insoluble Silk-II (β-sheet) proportionally increases [23].

**Figure 5:**
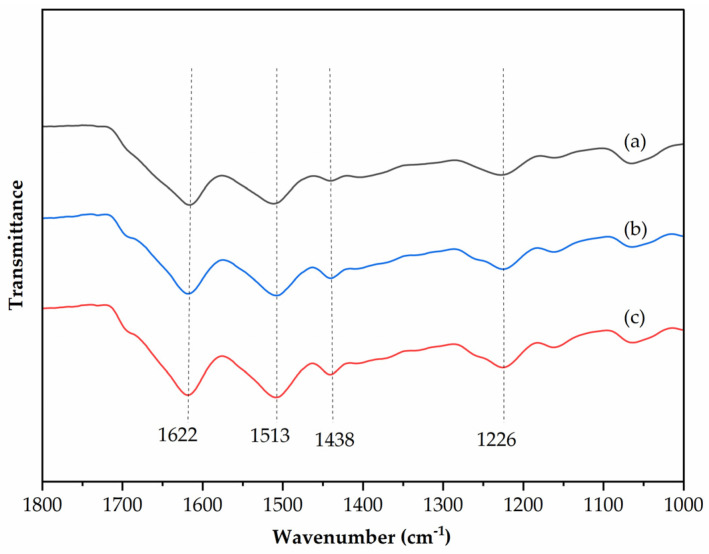
FTIR spectra of silk fibroin films prepared with different processes (**a**). Not degummed; (**b**) degummed from alkali boiling (80 °C, 30 min); (**c**) degummed from alkali steaming (100 °C, 120 min).

In the infrared spectroscopic analysis, Figure 5a of no degummed silk exhibits multiple characteristic absorption peaks closely associated with amide bands, with notable peaks at 1622 cm^−1^ and 1513 cm^−1^. The peaks at 1438 cm^−1^ and 1226 cm^−1^ indicate the presence of Silk-II (β-sheet), yet the α-helix structure remains dominant, resulting in steady overall transmittance, indicating structural stability. Additionally, the denser structure of raw silk enhances light absorption and scattering during transmission, thus reducing transmittance. Figure 5b, degummed from alkali boiling at a rate of 27%, reveals the further removal of sericin proteins and other non-fibrous components, leading to a further reduction in or complete disappearance of the Silk-I (α-helix) conformation. This is reflected in the further weakening of peaks at 1622 cm^−1^ and 1513 cm^−1^, alongside changes in the peaks at 1438 cm^−1^ and 1226 cm^−1^, indicating a continued increase in the proportion of Silk-II (β-sheet). Such drastic structural changes are manifested in the infrared spectrum as a further weakening or near disappearance of amide band absorption peaks, particularly at wavenumbers closely associated with the Silk-I (α-helix) conformation in raw silk. Figure 5c of alkali steaming with a degumming rate of 25% shows that the removal of some sericin proteins and other non-fibrous components has led to a certain degree of disruption in the Silk-I (α-helix) conformation, particularly at critical wavenumbers such as 1622 cm^−1^ and 1513 cm^−1^, indicating a reduction in α-helix structures. Changes in the peaks at 1438 cm^−1^ and 1226 cm^−1^ reflect an increase in the β-sheet structure. Furthermore, the looser structure of the silk fibers post-degumming facilitates greater light penetration, resulting in a significant increase in transmittance.

Overall, after degumming through alkaline boiling and steaming, as the degree of degumming increases, the fiber structure becomes more loose, and the transmittance increases; However, the characteristic peaks of the fabric at 3274, 1622, 1513, 1438, and 1226 cm^−1^ show little change, indicating that there is no significant difference in the secondary structure after degumming treatment, and that no new molecular structures or functional groups have been generated.

### 3.6. Degumming Effect on Pattern Appearance

Silk georgette exhibits changes in raw and degummed silk at different degumming rates. Raw silk shows better light transmittance and stiffness, while degummed silk presents a glossy, soft, and skin-friendly texture. The contrast between raw and degummed silk is most pronounced, with rich fabric texture resulting in distinctive local degumming patterns (i.e., alkali agent printing) [24]. The experiments demonstrate that both alkaline boiling and steaming processes, when controlled for different amounts of slaked lime, temperatures, and durations, can achieve various degrees of degumming in silk crepe fabrics, from no degumming to partial or full degumming. This section selects silk georgette with degumming rates of 0%, 10%, 15%, 20%, and 25% for dyeing and pattern development experiments as described in Section 2.2.4.

#### 3.6.1. Large Area Pattern Appearance

The depth of print color is a key indicator of the appearance of printed silk fabric products. Table 4 shows the color performance on the surface of silk crepe fabrics dyed with pomegranate peel at different degumming rates.

As shown in Table 4, the degumming rate significantly affects the color depth of silk crepe surfaces. The K/S value decreases from 17.97 for untreated raw silk to 0.90 for fully degummed silk. Raw silk fibers exhibit higher color absorption due to the more accessible amino groups in sericin, allowing for faster and greater dye uptake compared to fully degummed silk [25]. Furthermore, by increasing the degumming rate from 0% to 20%, the K/S value drops rapidly by about four to six units for every 5 percentage points. Beyond 20%, when the degumming rate reaches 25%, the K/S value only decreases by about 1.7 units, stabilizing as the residual sericin on the silk crepe surface decreases and the remaining fibroin dyeing becomes more stable.

**Figure 6:**
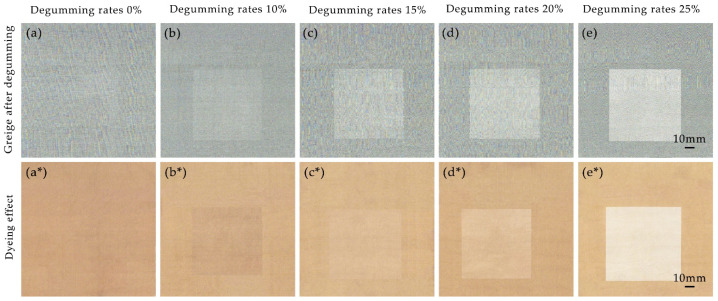
Effect of alkaline printing on georgette greige at different degumming rates (**a**–**e**) and comparison with pomegranate peel after dyeing (a*–e*).

Figure 6 illustrates the pattern appearance of alkali agent printing on silk georgette at different degumming rates, including both the untreated white fabric and the pomegranate peel-dyed fabric (with a deep background and light pattern). In these images, the raw silk serves as the background, while the degummed silk forms the patterned sections.

As the degumming rate increases from 0% to 10%, the silk georgette exhibits a subtle and distinctive dark pattern effect due to variations in lightness and darkness. When the degumming rate rises further to 25%, the pattern effect becomes more pronounced. The color on the raw silk background remains relatively stable, showing good translucency and stiffness, while the central patterned areas, made of degummed silk, show significant changes. As the degumming rate increases, the color of these areas gradually lightens. By the time degumming is complete, the fabric displays a glossy, soft, and skin-friendly texture with the most pronounced pattern effect and a richly textured fabric surface.

#### 3.6.2. Fine Pattern Appearance

The clarity of pattern outlines is another crucial indicator of the appearance of printed silk fabric products. To achieve a high-quality and detailed pattern effect, it is essential to prevent the bleeding of the pattern edges during the printing process. Although both alkaline boiling and steaming methods can achieve various degrees of degumming in silk georgette, there is a notable difference in pattern performance between these two methods. Alkaline steaming can produce finely detailed patterns by controlling the amount of starch at different mass fractions.

Table 5 presents data on the width of fine lines in alkali agent printing with different starch mass fractions, under conditions of complete degumming (saturated lime water paste, steam temperature 100 °C, steam time 120 min). The results indicate that all printed line widths are greater than the original width (1000 μm), with some pattern bleeding observed. This bleeding is related to the capillary effect of the fabric; after the alkaline paste is applied, free water infiltrates along the silk fibers, carrying hydroxide ions and causing pattern outlines to bleed [26].

**Figure 7:**
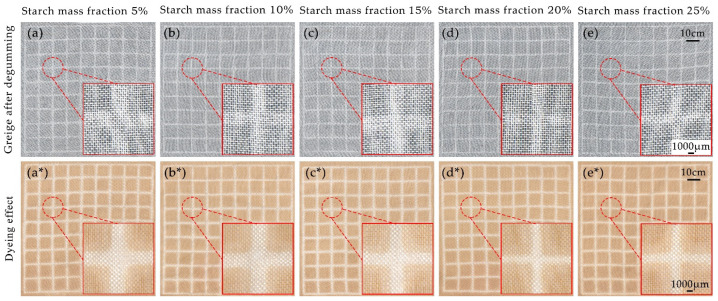
Effect of alkaline printing on georgette greige patterns at different starch mass fractions (**a**–**e**) and comparison of fineness quality after pomegranate peel dyeing (a*–e*).

Additionally, the flow properties of the paste are crucial for pattern clarity. Figure 7 shows the effects of different starch mass fractions on fine pattern printing and post-dyeing results. With starch mass fractions of 5%, 10%, and 15%, the paste exhibits high flowability and low viscosity, resulting in poor pattern clarity and significant bleeding. When the starch mass fraction increases to 20%, paste viscosity rises, and the line width approaches the original width with reduced edge spreading, yielding better pattern clarity and finer detail. Further increasing the starch mass fraction to 25% shows no significant change in line width.

Thus, under complete degumming conditions, a starch mass fraction of 20% provides the best clarity for alkali agent-printed patterns. Compared to the alkaline boiling method, alkaline steaming offers greater control over pattern definition and detail in printing.

## 4. Conclusions

The study analyzed alkali degumming of silk georgette fabrics using both alkali boiling and alkali steaming methods, focusing on the degumming and printing effects. By adjusting the amount of lime, the temperature, and the time conditions, both alkali boiling and alkali steaming can achieve varying degrees of degumming, from none to partial or complete degumming. During alkali boiling, the silk proteins are fully exposed to the alkali solution. At 80 °C for 30 min, the degumming rate reaches 27%. In contrast, alkali steaming relies on steam to slowly release the alkali from the paste, since the water has not yet reached its boiling point; even when alkali steaming for 120 min, the degumming rate remains consistently below 20%. Therefore, alkali boiling is rapid and effective, while alkali steaming requires a longer and slower process. This degumming control allows for changes in color depth in alkali-printed silk georgette. Under complete degumming conditions, the color depth, measured as a K/S value, is at its lowest and contrasts most strongly with the unprocessed raw silk, showing the most significant alkali printing effect. Compared to alkali boiling, alkali steaming, incorporating techniques such as screen printing and block printing, offers greater control over pattern freedom and detail. By leveraging the unique features of both degumming methods, traditional Chinese alkali printing techniques can be further expanded in contemporary silk degumming and printing, enhancing artistic expression and application.

## Figures and Tables

**Table 1:** Results of slaked lime concentration on degumming of silk georgette.

Slaked Lime Concentration/(g/L^−1^)	pH Value	Alkali Boiling Degumming Rate/%	Alkali Steaming Degumming Rate/%
0.05	11.00	11.32 ± 0.34	11.11 ± 2.75
0.10	11.32	16.57 ± 0.62	11.82 ± 2.13
0.20	11.50	24.51 ± 0.18	18.90 ± 1.52
0.30	12.13	27.17 ± 0.55	25.27 ± 0.54
0.40	12.50	27.43 ± 0.41	25.00 ± 0.45
0.50	12.55	28.13 ± 0.75	26.13 ± 0.62

**Table 2:** Results of different temperatures on degumming of silk georgette.

Temperatures/°C	Alkali Boiling Degumming Rate/% (30 min)	Alkali Steaming Degumming Rate/% (120 min)
60	20.28 ± 0.24	2.82 ± 2.11
70	24.54 ± 0.11	3.01 ± 1.17
80	27.12 ± 0.35	6.21 ± 1.46
85	31.47 ± 0.16	10.61 ± 2.62
90	34.01 ± 0.38	12.88 ± 1.31
95	38.35 ± 0.52	18.79 ± 1.05
100	42.14 ± 0.85	25.15 ± 0.26

**Table 3:** Results of different steam time on degumming of silk georgette.

Time/min	Alkali Boiling Degumming Rate/% (80 °C)	Alkali Steaming Degumming Rate/% (100 °C)
15	25.34 ± 0.56	16.54 ± 2.60
30	27.12 ± 0.35	20.00 ± 0.94
45	31.24 ± 0.81	21.43 ± 0.51
60	35.14 ± 0.62	22.53 ± 1.20
75	39.32 ± 1.17	23.72 ± 0.89
90	43.16 ± 0.62	24.41 ± 0.52
120	52.37 ± 0.54	25.27 ± 0.54
150	60.31 ± 0.78	25.54 ± 1.31

**Table 4:** Comparison of pomegranate peel-dyed color yield under printing with different degumming rates.

Degumming Rate	K/S Values	Specimen
0	17.97	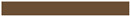
10	11.96	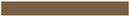
15	6.60	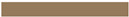
20	2.61	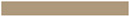
25	0.90	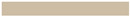

**Table 5:** Fineness of printing patterns with alkali agent under different mass fractions of starch.

Hydrated Lime Mass Fraction	Line Width
5	1555.53
10	1490.75
15	1386.58
20	1094.43
25	1080.28

## Data Availability

The data that support the findings of this study are available from the corresponding author upon reasonable request.
